# Gestalt’s Perspective on Insight: A Recap Based on Recent Behavioral and Neuroscientific Evidence

**DOI:** 10.3390/jintelligence11120224

**Published:** 2023-12-09

**Authors:** Mary Vitello, Carola Salvi

**Affiliations:** 1 Department of Psychology, University of California Los Angeles, Los Angeles, CA 90095, USA; mvitello@g.ucla.edu; 2 Department of Psychology and Social Sciences, John Cabot University, 00165 Rome, Italy; 3 Department of Psychiatry and Behavioral Sciences, University of Texas at Austin, Austin, TX 78712, USA

**Keywords:** insight problem-solving, “Aha!” moment, pupillometry, Gestalt, perception, attention, creativity, neurophysiology

## Abstract

The Gestalt psychologists’ theory of insight problem-solving was based on a direct parallelism between perceptual experience and higher-order forms of cognition (e.g., problem-solving). Similarly, albeit not exclusively, to the sudden recognition of bistable figures, these psychologists contended that problem-solving involves a restructuring of one’s initial representation of the problem’s elements, leading to a sudden leap of understanding phenomenologically indexed by the “Aha!” feeling. Over the last century, different scholars have discussed the validity of the Gestalt psychologists’ perspective, foremost using the behavioral measures available at the time. However, in the last two decades, scientists have gained a deeper understanding of insight problem-solving due to the advancements in cognitive neuroscience. This review aims to provide a retrospective reading of Gestalt theory based on the knowledge accrued by adopting novel paradigms of research and investigating their neurophysiological correlates. Among several key points that the Gestalt psychologists underscored, we focus specifically on the role of the visual system in marking a discrete switch of knowledge into awareness, as well as the perceptual experience and holistic standpoints. While the main goal of this paper is to read the previous theory in light of new evidence, we also hope to initiate an academic discussion and encourage further research about the points we raise.

## 1. Introduction

The scientific understanding of insight problem-solving originates from the Gestalt psychologists in the early 20th century. Before the Gestalts, the prevailing viewpoint posited that the human mind inherently establishes associations during trial-and-error learning, leading to a mode of reproductive thinking. When confronted with commonplace problems, individuals would merely reproduce solutions that they had previously correlated with successful outcomes by expanding, or modifying, their existing associations, implying the absence of genuinely novel creations (Thorndike 1911). Gestalt psychologists, instead, theorized that insight problem-solving unfolds through a paradigm of productive thinking. Within this framework, problem-solvers would overcome conventional associations and perceive problems through an entirely novel lens (Köhler 1925; Wertheimer 1959). These novel solutions emerge together with an abrupt sensation of apprehending, also termed an “Aha!” moment. For Gestalt psychologists, insight manifests as the transition from a state of uncertainty regarding the achievement of a problem’s objective to an in-depth comprehension of the problem itself and thus its attendant solution (Maier 1940) in an off–on matter, as a whole, or as “Gestalt”.

In his seminal work, Wolfgang Köhler (1925) documented a chimpanzee’s attempts to access out-of-reach bananas. Fortuitously, the chimpanzee managed to see the crates in its cage as potential building blocks for a makeshift staircase. By stacking and ascending the assembled crates, the chimpanzee successfully accessed the bananas. Köhler concluded that the chimpanzee’s reorganization of information in its visual field is what permitted the emergence of an insightful solution. The sudden off–on switch into awareness aligns with phenomena such as figure–ground reversals, in which “elements at one moment are seen as one unity, and at the same moment, another unity appears with the same elements” (Ellen 1982, p. 324). This perspective underscores the interplay between Gestalt’s problem-solving outlook and the foundational principles of Gestalt perceptual experience. This parallelism becomes especially cogent when a cognitive problem and its solution are provided with pictorial representations, in geometric or graph-theoretic forms. For example, this is the case of the problems discussed by Max Wertheimer (1959) in the book *Productive Thinking*. In such contexts, the discovery of a solution to a problem materializes as the emergence of an ordering or reordering between the elements in the pictorial representation, which (at an abstract level) is comparable to the emergence of a perceptual organization on an array of optical stimuli. Indeed, the Gestalt psychologists argued that perceptual experience is an active and dynamic process involving the mind’s inherent tendency to organize sensory information into coherent forms. To them, this process is not restricted to perception but expands also to the way in which we solve problems and how we experience the emergence of a solution as a whole (Köhler 1925; Wertheimer 1959).

Until recently, most of the academic discussions in support of, or in contrast to, the Gestalt theory on insight problem-solving have been based on behavioral studies. Those studies allowed fundamental steps forward in the cognitive understanding of problem-solving. However, it is thanks to neurophysiological results and new methodological paradigms, such as the use of self-reports when studying insight (Bowden et al. 2005; Kounios and Beeman 2009, 2014), that Neo-Gestalt theorists of insight (as termed by Weisberg 2018) have been able to ground with neurophysiological evidence the view of insight as *a special process*, which is more in line with its initial conception.

Further, considering the renewed interest in their theory (e.g., Mungan 2023) in this review, we aim to retroactively interpret some core points of the Gestalt psychologists on insight based on what we have learned from its study in the field of cognitive neuroscience. While Neo-Gestalt (or Neuro-Gestalt) theories provide a step forward in our understanding of insight problem-solving, a comprehensive review of this parallelism is still lacking.

We focus on three main points that were raised by the Gestalt theorists and read them considering novel evidence. *First* is the role of perceptual experience in problem-solving cognition. Was the parallelism between bistable figures and insight problem-solving warranted? *Second* is the holistic approach. What has recent research discovered about the idea that solutions to problems sometimes come to mind in an off–on manner? *Third*, while not explicitly, the Gestalt psychologists did assume that the solution to problems comes “with sudden clarity” (Köhler 1925; Wertheimer 1959). Can we see in this statement a proto-assumption that insightful solutions might be characterized by a perception of higher accuracy?

## 2. The Role of Perceptual Experience in Problem-Solving Cognition: Was the Parallelism between Bistable Figures and Insight Problem-Solving Warranted?

A critical link between perceptual experience and the physiological markers of insight problem-solving is provided by the study of pupil dilation. As we mentioned, one of the central ideas of the Gestalt psychologists was that the recognition of bistable figures, in terms of object interpretation, can rise suddenly following a reconfiguration of the visual constituents into a new, integrated Gestalt. Analogously, during problem-solving, a solution can unexpectedly emerge holistically, triggered by a reinterpretation of the constituent elements of the problem (Köhler 1925). Both instances entail a restructuring of the problem’s elements or figures, facilitating the emergence of a solution, or perception, into conscious awareness. This restructuring is phenomenologically marked by sensations of surprise, satisfaction, and pleasure, often articulated through the exclamation “Aha!” (Danek and Wiley 2017). When exposed to instances of perceptual and conceptual ambiguity, such as when confronted with bistable figures or attempting to unravel the solution to a problem, individuals tend to seek a recognizable structure within their perceptual or imaginative frameworks, akin to deciphering “connecting the dots” puzzles (Salvi et al. 2020). Undergoing an insight experience involves an underlying top-down subconscious reorganization of stimulus attributes, wherein the coherence of this configuration promptly engages conscious awareness (Salvi 2023).

Crucially, the question that arises pertains to whether this parallelism between perceptual experience and insight problem-solving is merely an illustrative analogy or whether the two share deeper commonalities. Nearly a century after Köhler’s investigations, research has unveiled that this parallel between visual perception and insight problem-solving is, indeed, grounded in markedly similar behavioral proxies as physiological correlates. Laukkonen and Tangen (2017) demonstrated that observing a bistable version of the Necker cube (vs. two alternating cubes) can lead to more insights when solving following verbal problems that require reorganization. In a similar vein, Bianchi and colleagues found that prompting individuals to “think in opposites” in visuospatial problems encouraged insights more than an overt hint at the problem (Bianchi et al. 2020). Specifically, the authors showed how the prompt to think in terms of opposites fosters a representational change in problem-solving by extending the search space. Together, these studies demonstrated cross-modal facilitation of perception to insight problem-solving.

When confronted with bistable figures, individuals undergo a phenomenon known as “perceptual rivalry”, wherein their visual perception oscillates between various potential interpretations, instead of remaining constant on a single one (e.g., as seen in the Necker cube effect). Neurophysiological studies have indicated that participants’ pupil diameter increases immediately before they declare engaging in perceptual reorganization (Einhäuser et al. 2008). Specifically, investigations have observed a rise in average pupil diameter to greater than baseline before conscious recognition of bistable visual stimuli (Einhäuser et al. 2008; Kietzmann et al. 2011).

Based on the above-mentioned results, Salvi et al. (2020) demonstrated that pupil size increased with a 60.5% likelihood in trials resolved through insight (with peak dilation occurring around 200 milliseconds before individuals declared experiencing an insight, i.e., during the “Aha!” moment). The change in pupil dilation was observed regardless of insight accuracy, corroborating the idea that false insights have the same phenomenology as accurate insights (Danek and Wiley 2017; Laukkonen et al. 2020). In this experiment, the authors demonstrated that the two switches (the figurative and the conceptual one) are both associated with the same “corollary” behavioral response (i.e., pupil dilation) and thus that the Gestalt hypothesis is valid.

Further, the observed increase in pupil dilation suggests a potential involvement of the locus coeruleus–norepinephrine (LC-NE) system in association with the “Aha!” experience. Pupil dilation serves as an indirect marker of noradrenergic activity, which is associated with creativity, cognitive flexibility in problem-solving, and the functional integration of the overall attentional brain system. Various studies have highlighted the role of noradrenergic activity in these processes (Beversdorf et al. 1999; Campbell et al. 2008; Corbetta et al. 2008; Coull et al. 1999; de Rooij et al. 2018; Sara 2009). In our cognitive system, attention and consciousness fulfill separate roles and are linked to distinct brain structures, but they maintain a pronounced interconnection (Koch and Tsuchiya 2007). Structures such as the LC and the amygdala play crucial roles in notifying and alerting frontal cortical regions to redirect ongoing processing toward the significance of new stimuli or concepts (e.g., Duncan and Barrett 2007). The LC-NE system, specifically, has a designated function of interrupting current functional networks and, by triggering a “reset” in target structures, fosters the development of new networks by redirecting attention (Sara and Bouret 2012). A similar redirection of attention toward a particular thought occurs when individuals experience an “Aha!” moment, in which an insightful idea suddenly breaks into their train of thought, refocusing their attention on a potential solution to a problem.

While further exploration is needed (and encouraged as a purpose of this review) to fully elucidate the implications of this physiological response in both perceptual and problem-solving tasks, so far these studies have substantiated Gestalt psychology’s conceptualization of insight problem-solving being akin to the reorganization of bistable figures. Moreover, they have provided evidence that the experience of insight is characterized by a non-continuous process, as the pupillary response could serve as an indicator of the transition from unconscious to conscious awareness (Laeng and Teodorescu 2002). While the outcomes of Salvi et al. (2020) have already been replicated by Becker et al. (2021), capturing the precise instant at which an idea materializes remains a multifaceted endeavor.

Thus far, it has been established that the shift in pupil size is observed approximately 200 milliseconds before individuals press a button to signify the occurrence of an “Aha!” moment. The variation in pupil size likely represents a physiological marker that may precede, follow, or coincide with the transition into awareness of the outcomes of unconscious processes (Salvi 2023). In summary, evidence from contemporary empirical work has demonstrated that the conceptual parallelism between ambiguous figures and insight problem-solving share physiological biomarkers, as well as a cross-modal facilitation of these two processes, suggesting a deeper link.

## 3. The Holistic Approach: What Has Recent Research Discovered about the Idea That Solutions to Problems Sometimes Come to Mind in an Off-On Manner?

The Gestalt School of Psychology was grounded in the idea that perceptual experiences are holistically organized, meaning that sensory stimuli are spontaneously organized into meaningful and holistic patterns rather than perceived as isolated elements.

Using Koffka’s (1935, p. 176) words: “The whole is something else than the sum of its parts, because summing is a meaningless procedure, whereas the whole-part relationship is meaningful”. Similarly, insight problem-solving is processed in a discrete off–on manner, and when solutions to problems emerge, they do so as a “whole”, and the solver cannot retroactively report the reasoning process that led him or her to the solution.

Metcalfe (1986) monitored the evaluation of participants regarding their proximity to arriving at a solution, measured as “warmth”. The findings revealed that, in the context of insight problems, the perception of warmth did not escalate until the final 10 s before the solution was reached, demonstrating how those solutions occur abruptly as a whole. In contrast, when dealing with analytic solutions, the warmth ratings demonstrated a more gradual increase over time. Additionally, Metcalfe investigated the types of responses that participants provided based on whether warmth ratings increased incrementally or suddenly. It was observed that responses connected to abrupt surges in warmth (indicative of insights) were more frequently correct compared to responses associated with gradual increments in warmth (representative of analytical problem-solving).

The neurophysiological findings documented in the problem-solving literature have consistently demonstrated the presence of two distinct levels of information processing when individuals generate ideas. The first level is characterized as continuous, explicit, and conscious, while the second level is discrete and implicit and operates below conscious awareness. This duality is reflected in the differentiation between problem-solving through analysis, which involves a gradual and explicit step-by-step approach to finding a solution, and problem-solving through insight, which involves a sudden shift in cognitive states, in which the solver transitions from a state of not knowing to a state of suddenly knowing the solution holistically (Jung-Beeman et al. 2004; Smith and Kounios 1996). Specifically, among other results, the intersection of multiple research techniques has revealed a sequence of events that has enabled scientists to pinpoint a specific moment occurring within the last 560 milliseconds before people report having an insight (including gamma activation over the right temporal lobe and pupil dilation) (see Salvi 2023 for a review; Jung-Beeman et al. 2004; Salvi et al. 2020). These findings have solidified the nature of insight problem-solving as a discrete off–on phenomenon also in terms of its accessibility to consciousness (Smith and Kounios 1996). When individuals solve problems through insight, indeed, they lack access to intermediate knowledge because this information is processed below the threshold of conscious awareness. Consequently, insights do not provide any intermediate results. Without meaningful potential solutions to guess, those who rely on insight processing tend to run out of time rather than make errors of commission. In contrast, step-by-step problem-solving unfolds gradually and within conscious awareness, allowing participants to access partial information on which they can base a guess before the response deadline. This process often leads to more errors of commission and a lower likelihood of timing out (Kounios et al. 2008; Salvi et al. 2016).

Further evidence of this all-or-none rise of the problem solution was provided by Laukkonen et al. (2021), who used a dynamometer to track the intensity of the insight experience. Their results showed that participants instinctively (i.e., without explicit instruction) exerted greater pressure on the dynamometer in a single slope of pressure (as a whole) during “Aha!” Experiences, and the magnitude of the “Aha!” experience corresponded to the accuracy of the solutions (see the final section for a discussion of accuracy).

Although it is challenging to capture the shift into awareness that characterizes an insight, researchers have been able to utilize advancements in techniques to identify physiological measures that might overlap with insight emerging into awareness. As mentioned above, during both perceptuals and conceptuals associated with having an insight, the pupils dilate (Einhäuser et al. 2008; Salvi et al. 2020), and pupil dilation has been argued to be a proxy for the switch from unconscious to conscious states (Bijleveld et al. 2009; Chapman et al. 1999). That said, insights are ineffable; capturing the exact instance when the ideas burst into awareness might be ambitious at this time and with the current techniques, but it is worth posing this question to encourage future investigation.

The premise of holistic perceptual experiences was of keen interest to the Gestalt School of Psychology. Indeed, the all-or-none quality of the perception of bistable figures grounded the Gestalts’ perspective on insight problem-solving as a comprehensive experience. Contrary to the process of solving problems in an analytical, stepwise fashion, insights are characterized by their sudden appearance into awareness as a whole. Recent methodological advancements have begun to reveal physiological indicators of the sudden awareness associated with insight problem-solving. While capturing the precise moment at which an insight enters awareness remains a challenging endeavor with present methodologies, these advancements provide evidence of a subjective, as well as a physiological, indication of a holistic switch when an insight solution is found.

## 4. The Gestalt Psychologists Assume That the Solution to Problems Comes “With Sudden Clarity.” Can We See in This Statement a Proto-Assumption That Insightful Solutions Might Be Characterized by a Perception of Higher Accuracy?

When confronted with a question, a natural inclination might be to think step-by-step about problem elements to obtain a solution (Danek 2018). However, in cases of insight, this effortful strategy is absent, and a solution springs to mind with clarity and conviction about its correctness (Danek and Wiley 2017). Why should we trust such thoughts that have no accessible preceding analytical steps? In this last section, we discuss insights in terms of their adaptive function to select the simplest and most fitting solution and how this solution might be captured by a neurocomputational theory of insight (Laukkonen et al. 2023).

The hallmark of insight, according to Gestalt theory, is its suddenness and clarity. People experience a sudden shift in understanding, often warranted by problem-solving accuracy (Danek and Wiley 2017; Salvi et al. 2016; Laukkonen et al. 2021; Webb et al. 2016). A validated line of research has demonstrated that insights tend to be more accurate, and this accuracy holds across several different task domains: compound remote associates problems (CRAs; Salvi et al. 2016; Laukkonen et al. 2021), anagrams (Salvi et al. 2016), rebus puzzles (Salvi et al. 2016), line drawings (Salvi et al. 2016), and magic tricks (Danek et al. 2014; Hedne et al. 2016).

When considering the subjective, affective experience of insight, “correct solutions bring about a sensation of closure and satisfaction” (Danek and Salvi 2018, p. 485). Conversely, in the case of incorrect solutions, certain elements might be absent or fail to harmonize, leading to an incomplete sense of the Gestalt. This divergence is also evident in the subjective assessments of solvers’ solution experiences: Danek and Wiley (2017) demonstrated that correct solutions elicit a more pleasurable feeling than incorrect ones. This experience of achieving a Gestalt, followed by pleasure, bears similarity to comprehending jokes and metaphors. Analogous to grasping a joke, gaining insight involves delving into alternative meanings and concepts that then suddenly align into a unified whole, triggering the “Aha!” moment (or a burst of laughter). Notably, neuroscientific investigations reveal that the brain circuitry implicated in insight is also pivotal for recognizing remote semantic relationships, metaphors, and alternate meanings (for a review, see Kounios and Beeman 2014).

The Gestalt school noted the proclivity for humans to perceive complex sensory information in the simplest, most meaningful, and most complete way. In simple terms, the law of *Prägnanz* is a case of cognitive parsimony: a principle asserting that our cognitive systems prefer economical and elegant representations of reality (Koffka 1935; Wertheimer 1923). Sudden insights exemplify this principle, as they succinctly encapsulate the most pertinent and likely solution. A recent study supports this conclusion: Korovkin et al. (2021) designed an experiment using the 10-penny problem. This problem has two types of correct solutions: one that forms a symmetrical (holistic) Gestalt and the second, which does not fit into simple schemes or symmetric forms. Their results demonstrated that symmetrical (holistic) solutions have a higher subjective rating of both the “Aha!” experience and the “feeling of elegance” than asymmetrical solutions. According to the authors, the “holistic solutions which are presumably encoded by schemes lead to greater certainty about the correctness of the answer, since the scheme allows one to trace a path to a goal state within the mental lookahead” (Korovkin et al. 2021, p. 623). As Danek and Kizilirmak put it, “Essentially, Korovkin et al. (2021) demonstrate that the “Aha!” experience is determined by features of the solution—and not by features of the problem. Although the problem remained the same, the resulting solution experience, measured by a number of rating scales […], differed, depending on which type of solution was found” (Danek and Kizilirmak 2021, p. 610).

The finding that insight solutions tend to be more correct than those without insight bears on important questions about a possible adaptive nature of insights (Salvi 2023; Laukkonen et al. 2023).

One way to elucidate the processes involved in insight is through the purview of predictive processing, which is based on the intuitive idea that surprise governs learning (Friston et al. 2017; Laukkonen et al. 2023). In simple terms, this perspective takes that, because the brain does not have unlimited access to the information in the external environment, it must create a cognitive model based on inferences (Friston 2009). When our predictions of a world state are incorrect, we then update our models of the world to support adaptive behavior. Correction of false inferences—or prediction errors—is important for model updating to refine beliefs and expectations (Feldman and Friston 2010). Crucially, in the context of the sudden rise of an insight into awareness, Bayesian model reduction may be involved in the identification of the most parsimonious and best-fitting solution to a problem, as argued by Laukkonen et al. (2023). This goal is achieved via a restructuring among someone’s existing hypotheses, explanations (Friston et al. 2016a, 2016b), or initial representation of the problem. This Bayesian model selection is an act of discrete processing that permits a restructuring to take place, ultimately resulting in a discovery at a higher-order level of sentience (Friston et al. 2017).

This notion finds resonance in physiological processes, in which the minimization of model error is mediated by neurotransmitters, such as dopamine (Feldman and Friston 2010; Haarsma et al. 2021). In this way, a sudden insight might arise when the amalgamation of previously separate and loosely related pieces of information is selected as a coherent and parsimonious solution. The feelings of pleasure and confidence immediately after insight is realized may also be captured by the dopaminergic signaling that occurs during prediction errors (Tik et al. 2018; Oh et al. 2020; Salvi et al. 2015, 2021). The moment of insight is associated with increased activation in brain networks relating to salience signaling (Kounios et al. 2006; Kounios and Beeman 2009, 2014; Becker et al. 2021). In this way, dopamine signaling may be integral to the heightened confidence and affective experience of emerging insight (Danek and Wiley 2017; Laukkonen et al. 2023; Salvi 2023).

These findings complement the behavioral and neurophysiological literature discussed in the previous sections. On the observational level, individuals demonstrate behaviors, such as gaze aversion, pupil dilation, and increased frequency and duration of eye blinks (Salvi et al. 2015, 2020; Salvi and Bowden 2016). This disengagement of external attentional processing is thought to encourage the integration of conceptual disparate thoughts, allowing for an insight to emerge. Nevertheless, until recently, neurocomputational perspectives to explain the processes by which the brain can integrate information into a previously unsolved problem have been lacking. Implicit reorganization via a reduction in prediction errors, in the absence of new visual inputs, provides an apt framework to understand the behavioral, neurocomputational, and phenomenological experience of insight problem-solving (Laukkonen et al. 2023).

In summary, the characteristics of insight problem-solving, to be at once holistic, sudden, and more accurate than non-insight solutions, have perplexed researchers since the Gestalt psychologists initially formulated their perspectives on the topic. The feelings of clarity, reward, and satisfaction that accompany insights pose the question: why do we trust these sudden insights so confidently? Here, we provide some preliminary answers to this question. Recent advances in neurocomputational research have shed light on how information can be reconfigured into a holistic solution that appears suddenly, without subjective effort or visual or external cues. By reducing prediction errors by internal inferential strategies, insights may rise to awareness. Further, the feelings of reward associated with an “Aha!” moment can be characterized by the implication of dopaminergic signaling associated with prediction errors within this framework.

## 5. Conclusions and Future Directions

The Gestalt psychologists introduced a novel perspective on problem-solving conception. In lieu of the prevailing view by which learned associates dictate the success of solution finding, they advanced the notion that solutions can arise from a sudden and holistic restructuring of problem elements, similar to the way in which bistable figures are holistically recognized. However, the extent to which these parallels between perceptual experience and problem-solving provided a useful comparison, or instead illuminated something more critical about how information is processed more generally, has been debated for a long time (e.g., Weisberg and Alba 1981; Weisberg 1986). In recent years, advancements in cognitive neuroscientific techniques have begun to provide further evidence to answer these questions. This integration of phenomenologically inspired observations (such as those of Gestalt Psychologists, as well as recent developments discussed in the previous sections of this article) and cognitive neuroscience has illuminated the multifaceted nature of insight problem-solving and its underlying cognitive and neural processes.

Both the recognition of bistable figures and the sudden rise in insight into awareness are associated with an increase in pupil dilation. This marker is diagnostic of insights; thus, we could also use it to study insight when self-reporting of “Aha!” experiences is possible (for example with children or primates) (Salvi 2023). Further, the results of eye movement and EEG studies have led to the proposal that insights require a sensory-gating process to pull attention from the external environment toward internally oriented cognition (for a review see Kounios and Beeman 2009, 2014; Salvi 2023).

What is the role of crowded and uncrowded visual environments in insight problem-solving? Can this knowledge help us to find ways to facilitate insight occurrence? Further, does this physiological response signify a pivotal temporal juncture in the shift to conscious awareness? Is this small temporal window the instant when an idea switches into awareness? Can we draw deeper conclusions from what is, so far, only speculation?

Recent neurocomputational perspectives have advanced knowledge about how, in the absence of further visual or external input, a holistic reconfiguration of information emerges as a sudden insight. Here, we have tried to highlight how insights embody the concept of cognitive parsimony, integrating complex information into coherent and succinct solutions. Evidence from both subjective reports and physiological data have begun to illuminate the time course of insight problem-solving and reveal its discrete manner. Unlike analytical problem-solving, we have highlighted evidence of insight problem-solving emerging into awareness in an off–on, holistic manner. This approach harkens back to the Gestaltist principle of *Prägnanz*: humans prefer simple and recognizable forms of information. While the Gestalt psychologists primarily focused on visual forms, we have extended this understanding to the conceptual level. Insights carry with them feelings of certainty, clarity, reward, and satisfaction (Danek and Wiley 2017; Webb et al. 2016), posing interesting questions about why we trust these insights with such conviction. They spring to mind without any conscious effort or awareness, yet we are confident about their accuracy. By integrating neurocomputational perspectives (Laukkonen et al. 2023; Friston et al. 2017) with behavioral and physiological indicators of insight problem-solving, researchers have been granted a deepening understanding of the phenomena along levels of analysis. In the absence of new information, is the brain capable of integrating extant knowledge into new configurations to encourage insightful solutions?

As we navigate this juncture of century-old theories and modern cognitive neuroscientific evidence, several promising avenues for future research emerge. For example, while much evidence points toward its involvement, the particular role of dopamine and its associated circuits remain unclear. Along these lines, the parameters for which a solution is selected are underspecified, and computational models could address this issue with normative models of decision making.

In summary, we have traced the influence of the Gestalt psychologists on modern conceptions of insight problem-solving. This synthesis between historical tenets of Gestalt psychology and contemporary cognitive neuroscience underscores the multifaceted nature of insight problem-solving. By encouraging interdisciplinary approaches, they hopefully hold the potential to illuminate the intricate interplay among perception, cognition, and insight problem-solving.

## Data Availability

Not applicable.
